# Additive Value of Rheumatoid Factor Isotypes in Sjögren’s Syndrome Patients with Joint Complaints of Different Etiologies—Can Rheumatoid Factor IgA Serve as an Early, Poor Prognostic Biomarker Candidate?

**DOI:** 10.3390/ijms26104797

**Published:** 2025-05-16

**Authors:** Zsófia Aradi, Bernadett Bói, Gábor Nagy, Péter Antal-Szalmás, Kincső Mezei, Ildikó Fanny Horváth, Antónia Szántó

**Affiliations:** 1 Division of Clinical Immunology, Institute of Internal Medicine, Faculty of Medicine, University of Debrecen, 22 Móricz Zsigmond Street, H-4032 Debrecen, Hungary; aradi.zsofia@med.unideb.hu (Z.A.); mezei.kincso@med.unideb.hu (K.M.); fanny.horvath@med.unideb.hu (I.F.H.); 2 Gyula Petrányi Clinical Immunology and Allergology Doctoral School, University of Debrecen, Egyetem tér 1, H-4032 Debrecen, Hungary; 3 Department of Public Health and Epidemiology, Faculty of Medicine, University of Debrecen, Kassai út 26, H-4028 Debrecen, Hungary; boi.bernadett@med.unideb.hu; 4 Department of Laboratory Medicine, Faculty of Medicine, University of Debrecen, Nagyerdei krt 98, H-4032 Debrecen, Hungary; nagy.gabor@med.unideb.hu (G.N.); antalszp@med.unideb.hu (P.A.-S.)

**Keywords:** Sjögren’s syndrome, polyarthritis, rheumatoid arthritis, rheumatoid factor isotypes, rheumatoid factor, biomarker

## Abstract

The aim of the paper was to characterize rheumatoid factor IgA, IgG, and IgM isotypes in patients with Sjögren’s syndrome (SS) subsets, based on the absence or presence of joint complaints of different etiologies. In total, 164 SS patients were grouped based on whether they had polyarthritis as an extraglandular manifestation (*n* = 73, SS+pa), rheumatoid arthritis as an associated autoimmune disorder (*n* = 46, SS+RA), or Sjögren’s syndrome without inflammatory joint pain (*n* = 45, SS). The highest IgA rheumatoid factor isotype levels were detected in SS patients, whereas the lowest levels were found in the SS+RA group, without a significant difference. Neither IgG nor IgM RF differed significantly between the patient subclasses. In addition to other disease-specific markers, seropositive patients who were seropositive for any RF isotype were significantly more frequently anti-Ro/SS-A and anti-La/SS-B positive and had higher ESSDAI levels. In SS and SS+pa patients, a strong negative correlation was observed between IgA RF and age, whereas a strong positive correlation was found between IgA RF and ESSDAI, RF, IgA, IgG, anti-Ro/SS-A, and anti-La/SS-B levels. High total IgG levels together with high IgA RF levels occurred most frequently in SS patients (*p* = 0.05), whereas the combination of normal IgG and high IgM RF was significantly more frequent in the SS+RA group. The co-occurrence of high total IgG and normal IgM RF did not differ significantly between the patient subsets; however, this was the combination with the highest sensitivity (94.5%) for SS+pa patients. Based on our findings, rheumatoid factor isotypes have an additive value in the differentiation of non-erosive polyarthritis and erosive rheumatoid arthritis during the disease course of patients with Sjögren’s syndrome. All rheumatoid factor isotypes predict a more severe disease course, but IgA RF may serve as a candidate for being an early, poor prognostic factor for SS patients.

## 1. Introduction

Sjögren’s syndrome (SS) is a systemic autoimmune disease affecting primarily the exocrine glands, leading to the clinical picture of “sicca syndrome”. Besides these glandular symptoms, other organs might become involved, resulting in extraglandular manifestations (EGMs) [1]. Articular involvement is among the most common extraglandular manifestations. Approximately 30–60% of primary SS patients suffer from joint complaints, which are usually associated with multisystem involvement [2]. In our earlier study, we characterized the demographic, clinical, and laboratory parameters of SS patients with different kinds of inflammatory joint impairment to identify potential factors that help in distinguishing them. Among the laboratory parameters, we noticed that a high positive (≥3× upper level of normal) rheumatoid factor (RF) increased the chance of having associated rheumatoid arthritis (RA) compared to SS patients lacking joint issues. At the same time, high immunoglobulin G (IgG) levels showed lower odds of having RA in SS patients [3].

Rheumatoid factors are antibodies directed against the Fc portion of immunoglobulin G. Although predominantly encountered as IgM, rheumatoid factor can be composed of any isotype of immunoglobulins. RF testing in RA patients has been reported to have a sensitivity from 60% to 90% and a specificity of 85%. Rheumatoid factor (RF) levels can be elevated not only in RA but also in other autoimmune and non-autoimmune diseases [4,5].

The aim of this study was to investigate different RF isotypes (IgM RF, IgA RF, and IgG RF) in patients with SS alone, SS complicated with polyarthritis (pa) as an EGM (SS+pa), and SS associated with rheumatoid arthritis as a second autoimmune disorder (SS+RA) to find out whether differences between RF isotype patterns of the groups could be potentially useful in everyday practice. We also investigated whether there is a correlation between immunoglobulin isotypes and RF isotypes.

## 2. Results

### 2.1. Demographics

The male/female ratio was 3/42 in the SS group, 2/71 in the SS+pa subset, and 1/45 in the SS+RA subset. The mean age of the patients was the highest in the SS+RA group and the lowest in the SS group, but the difference was not significant among the three groups. No significant difference was observed in disease duration (Table 1).

### 2.2. Laboratory Parameters

#### 2.2.1. Basic Laboratory Parameters

No significant difference was found between the three patient groups regarding IgA, IgM, C-reactive protein, and rheumatoid factor levels. Remarkably, the highest IgG levels were measured in the SS group and the lowest levels in the SS+RA group, and the difference was significant between the three subsets (Table 1).

#### 2.2.2. Rheumatoid Factor Isotypes

**Figure 1:**
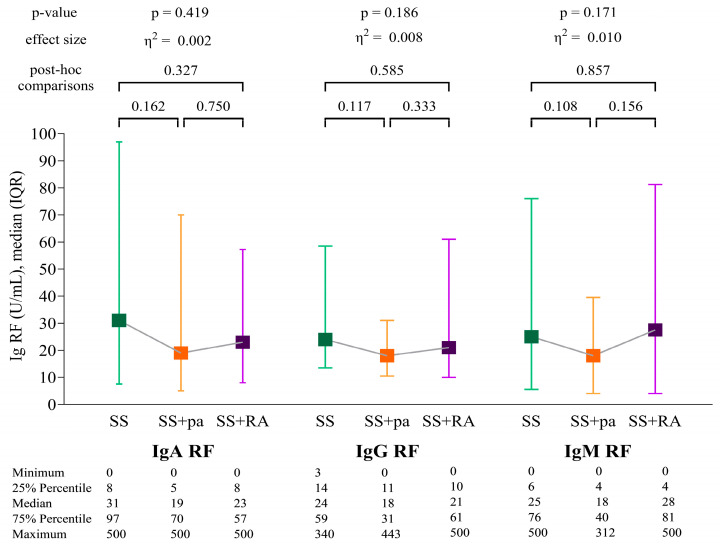
IgA RF, IgG RF, and IgM RF levels of the patient groups. Abbreviations: SS: Sjögren’s syndrome patients without joint pain; SS+pa: Sjögren’s syndrome patients with polyarthritis; SS+RA: patients with associated Sjögren’s syndrome and rheumatoid arthritis; IgA RF: immunoglobulin A isotype of rheumatoid factor; IgG RF: immunoglobulin G isotype of rheumatoid factor; and IgM RF: immunoglobulin M isotype of rheumatoid factor.

Among RF isotypes, the most notable difference between the three patient groups was found for IgA RF, with the highest levels in the SS group and the lowest in the SS+RA subgroup; however, the difference was not statistically significant. No significant difference was found between the patient groups when comparing IgG RF and IgM RF levels either (Figure 1).

#### 2.2.3. Correlations Between Patient Groups, Rheumatoid Factor Isotypes, and Disease-Specific Parameters

**Figure 2:**
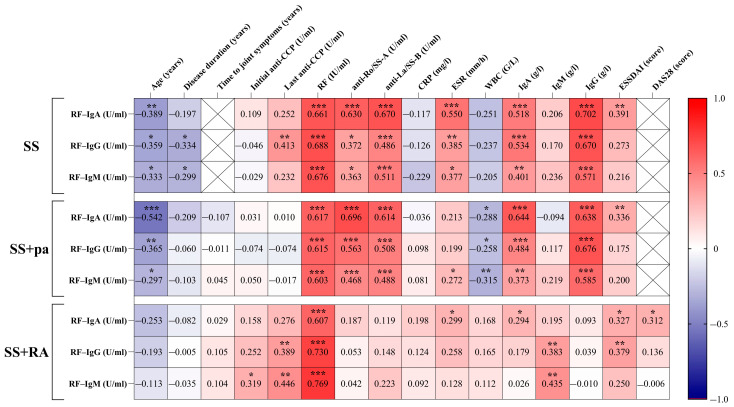
Correlation matrix of the relationship between RFs Ig classes and disease-specific parameters for each patient group. The relationship between the variables is shown by Spearman’s correlation coefficient (r_s_). * *p* < 0.05, ** *p* < 0.01, *** *p* < 0.001. Abbreviations: SS: Sjögren’s syndrome patients without joint pain; SS+pa: Sjögren’s syndrome patients with polyarthritis; SS+RA: patients with Sjögren’s syndrome associated with rheumatoid arthritis; RF: rheumatoid factor; anti-Ro/SS-A: anti–Sjögren’s-syndrome-related antigen A antibody; anti-La/SS-B: anti–Sjögren’s-syndrome-related antigen B antibody; ESSDAI: EULAR Sjögren’s syndrome disease activity index; anti-CCP: anti-cyclic citrullinated peptide antibody; CRP: C-reactive protein; ESR: erythrocyte sedimentation rate; WBC: white blood cell; DAS28: Disease Activity Score-28 for Rheumatoid Arthritis; IgA RF: immunoglobulin A isotype of rheumatoid factor; IgG RF: immunoglobulin G isotype of rheumatoid factor; IgM RF: immunoglobulin M isotype of rheumatoid factor.

The correlation matrix is shown in Figure 2.

Examining the relationship between the RF Ig subclasses and disease-specific parameters in the different patient subsets, significant negative correlations were found between age and each RF isotype in each patient group. Regarding disease duration, only IgG and IgM RF isotypes correlated significantly and negatively in control SS patients. Both the initial and the latest anti-cyclic citrullinated peptide (CCP) levels showed significant positive correlation with IgM RF results, but only in SS+RA patients. Lastly, anti-CCP levels and IgG RF levels showed a significant positive correlation in SS and SS+RA patients. As expected, all RF isotypes showed significant positive correlation with rheumatoid factor concentrations in each patient group. Unlike in SS+RA patients, the other two subsets showed a significant positive correlation between all RF isotypes and anti-Ro/SS-A and between anti-La/SS-B and total IgA and IgG levels. IgM levels correlated only with the IgG RF and IgM RF levels of SS+RA patients. Regarding erythrocyte sedimentation rate (ESR) values, significant positive correlation was observed in the SS group with each RF isotype, while SS+pa patients had a positive correlation with IgM RF and SS+RA patients with IgA RF levels. Interestingly, white blood cell count correlated negatively with all RF isotypes in SS+pa patients. The disease activity score obtained in 28 joints (DAS28) was calculated only in the SS+RA group; however, it correlated with IgA RF levels. Regarding the European Alliance of Associations for Rheumatology (EULAR) Sjögren’s syndrome disease activity index (ESSDAI), the most pronounced positive correlations were found with IgA RF; however, the IgG RF values correlated with the ESSDAI values in SS+RA patients as well.

#### 2.2.4. Differences Among Patient Groups According to the Negative or Positive RF Isotype Results

When grouped based on whether IgA, IgM, and IgG RF levels were below or above the cut-off value (denoted as negative or positive accordingly), there was no significant difference in any patient subsets (SS vs. SS-pa vs. SS+RA). However, regarding all isotypes, seropositive patients were significantly younger. Moreover, disease duration of positive IgA RF patients was significantly shorter than that of negative ones. ESSDAI was higher in each seropositive patient group regardless of RF isotype. We also noticed that positive testing for initial anti-CCP, RF, anti-Ro/SS-A, anti-La/SS-B, antinuclear antibody (ANA) and high ESR values were significantly more frequent in all positive IgA, IgM, and IgG RF patients than in the negative ones. In addition, IgG and IgM RF seropositive patients tested positive for anti-CCP significantly more often. Except for total IgM levels in positive IgA RF patients, all other immunoglobulin levels in all other positive RF isotype groups were significantly higher than in seronegative patients (Table 2).

Regarding the effect of ongoing immunomodulant or immunosuppressive medications on rheumatoid factor isotype levels, no significant difference was found among the frequency of drugs used and the occurrence of RF isotypes, with the only exception being sulfasalazine, used significantly less frequent in positive IgG RF patients compared to negative ones (Table 3).

#### 2.2.5. RF Isotype Levels According to the Occurrence of Extraglandular Manifestations and Associated Autoimmune Diseases

**Figure 3:**
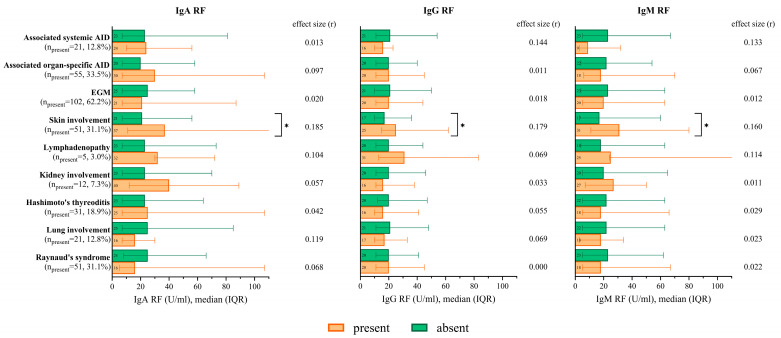
IgA RF, IgG RF, and IgM RF levels in cases of extraglandular manifestations and associated autoimmune diseases. * *p* < 0.05. Reference range of laboratory parameters: IgA RF < 20 U/mL, IgG RF < 20 U/mL, IgM RF < 20 U/mL. Abbreviations: IgA RF: immunoglobulin A isotype of rheumatoid factor; IgG RF: immunoglobulin G isotype of rheumatoid factor; IgM RF: immunoglobulin M isotype of rheumatoid factor; AID: autoimmune disease; and EGM: extraglandular manifestation.

If Sjögren’s syndrome-associated skin manifestations (purpura and cutaneous vasculitis) were present, significantly higher RF levels were observed regarding each isotype. In the case of further extraglandular manifestations or associated organospecific (Hashimoto’s thyroiditis, primary biliary cholangitis, and autoimmune hepatitis) or systemic (systemic lupus erythematosus and antiphospholipid syndrome) autoimmune diseases, there was no significant difference in RF isotypes (Figure 3). Among the associated autoimmune diseases (AID), only Hashimoto’s thyroiditis occurred frequently enough to be worth depicting separately.

#### 2.2.6. RF Isotypes in the Case of Low/Normal or Elevated Immunoglobulin Levels

**Figure 4:**
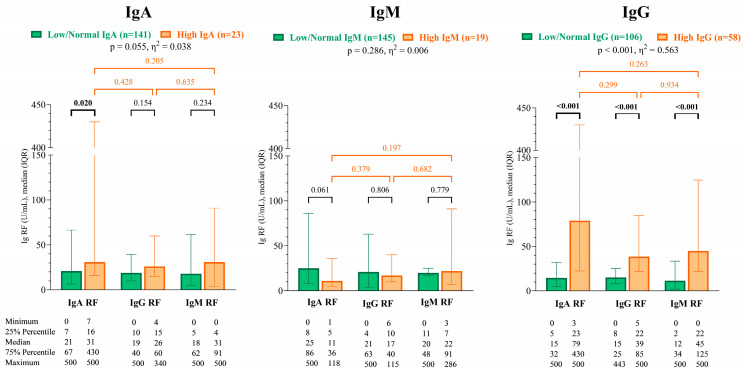
IgA RF, IgG RF, and IgM RF levels of the IgA, IgM, and IgG groups. Reference range of laboratory parameters: IgA: 0.7–4 g/L; IgG: 7–16 g/L; IgM: 0.4–2.3 g/L. Abbreviations: IgA RF: immunoglobulin A isotype of rheumatoid factor; IgG RF: immunoglobulin G isotype of rheumatoid factor; and IgM RF: immunoglobulin M isotype of rheumatoid factor.

The patients were also grouped according to whether their total IgA, IgM, and IgG levels were low/normal or high, and then, their RF isotypes were compared. In patients with high total IgG levels, all RF isotypes were significantly higher. IgA RF levels were significantly higher in patients with high total IgA levels. Such a phenomenon was not observed in the total IgM levels (Figure 4).

#### 2.2.7. Combination of Immunoglobulin and RF Isotype Qualitative Results and Their Occurrence in Patient Groups

Taking the above-mentioned findings together, the IgG results and certain RF isotypes were paired to see whether these combinations might help differentiate between the three groups. High total IgG levels, together with high IgA RF levels, occur most frequently in SS patients (*p* = 0.05), whereas the combination of normal IgG and high IgM RF is significantly more frequent in the SS+RA group. The co-occurrence of high total IgG and normal IgM RF did not differ significantly between the patient subsets; however, this was the combination with the highest specificity (94.5%) for SS+pa patients (Table 4).

## 3. Discussion

Sjögren’s syndrome is diagnosed eight times more often in women than in men, primarily around perimenopause, as supported by our cohort, as well as Ref. [6].

The pathogenesis of SS is multifactorial, and both the innate and the adaptive immune responses are affected. Recently, the role of Th17 cells has been suggested [1], and growing evidence support the fundamental role of vitamin D hypovitaminosis in the pathogenesis of autoimmune diseases [7]. Vitamin D is able to control the microbiome, balance the interleukin 31/33 axis, and consequently, improve immune tolerance [8]. Human leukocyte antigen (HLA) G-expressing cells are known to contribute to immune homeostasis by creating a milieu that attempts to control the processes responsible for the loss of self-tolerance during the development of autoimmune disorders [9].

Articular involvement is one of the most common extraglandular manifestations in Sjögren’s syndrome, which manifests primarily in the forms of arthralgia and non-erosive polyarthritis. Nevertheless, it is often associated with rheumatoid arthritis, which can lead to severe joint destruction and bone erosion. Therefore, it is important to distinguish non-aggressive, non-erosive manifestations from early rheumatoid arthritis. In our recent paper, we found that highly positive anti-CCP positivity improves diagnostic accuracy when it comes to diagnosing RA in a SS patient with long-standing polyarthritis [3]. The distinction can be further verified by the examination of RF isotypes.

As we know, B-cell hyperactivation is a hallmark of pSS, including polyclonal hypergammaglobulinemia, including the rheumatoid factor, antinuclear autoantibodies, and other autoantibodies, which are essential players in the development of systemic manifestations [10]. From our study, we can see that total IgG levels are highest in the SS group, significantly higher than in any of the other two patient groups, and still higher among polyarthritis patients than in the RA-associated group. Based on these results, we can see that higher IgG levels are inherent in Sjögren’s syndrome, and they are not caused by rheumatoid arthritis.

When examining the concentrations of RF isotypes between the patient groups, we noticed that the IgA-RF is the highest in the SS group and the lowest in the SS+RA subgroup, although the difference is not significant. This suggests that IgA isotype RF is more a marker of Sjögren’s syndrome than of rheumatoid arthritis. According to another study, serum concentration of IgA RF in patients with SS without EGMs was significantly higher than in patients with SS associated with RA, while in the latter, the IgM-RF isotype was more dominant [8]. These findings correspond to our results. In that paper, IgG-RF and IgA-RF presented higher concentrations in SS patients without EGM than in SS with EGM [11]. Our cohort presented similar results, although the differences were not significant. Lee et al. published even more comparable results as follows: the presence of IgA RF in patients with SS was associated with a significantly worse exocrine function and active serologic profile, and there was no association between IgA RF and EGM [12]. Regarding the active serologic profile, we found the same association. That said, in EGMs, although we did not find a significant difference in IgA RF, IgG RF, and IgM RF levels in most extraglandular manifestations, all RF isotypes were significantly higher if skin manifestations occurred.

Based on our results, RF levels have a strong positive correlation with each RF isotype, and so did IgG, anti-Ro/SS-A, and anti-La/SS-B levels, except for the SS+RA group. Our data correspond with the findings of Maślińska et al., who reported that RF-IgA showed the best diagnostic accuracy for SS and it correlated with anti-Ro/SS-A and anti-La/SS-B antibodies, even more closely than IgM RF. IgA RF may be considered an additional marker of immunological activity in SS, as its presence correlated with level of antibodies, which are characteristic for a serologic profile of SS [13].

The numerous shared features of all RF isotypes, especially for SS patients without RA-positive correlations with IgA, IgG, anti-Ro/SS-A, and anti-La/SS-B levels, lead us to the conclusion that these parameters are common indications of the polyclonal B-cell activation typical in SS [10], and they become less pronounced when RA co-occurs.

In a paper with the purpose of defining the diagnostic accuracy of RF isotypes in the clinical scenario of inflammatory arthralgia, where the differential diagnoses included lupus, SS, and osteoarthritis, RF IgM isotype and anti-citrullinated protein antibodies were more useful in confirming RA diagnosis than other RF isotypes [14]. Others found that the combined presence of IgG, IgA, and IgM isotype RF is characteristic of RA, while the presence of IgG and IgA isotype RF, in addition to the absence of the IgM isotype, is characteristic of other autoimmune diseases [15]. Van Hoovels et al. noticed that the combined positivity of RF IgM, IgA, and ACPA IgG had the highest specificity for RA diagnosis and classification [16]. In our cohort too, IgM RF alone was not enough to differentiate between patient groups. However, when combined with normal total IgG levels, high IgM RF could better define SS+RA patients.

As highlighted in a review paper, the presence of the IgA isotype of not only RF but also anti-modified protein antibodies indicates the key role of these antibodies in the inflammatory and destructive processes of RA. Furthermore, this isotype supports the mucosal origin hypothesis of the disease [17]. Our research revealed that the higher the IgA isotype RF, the more active the disease is based on the ESSDAI [18] and DAS28 [19], both for RA and Sjögren’s syndrome with or without polyarthritis. Based on these results, elevated IgA RF levels predict a more active disease and might serve as an unfavorable prognostic factor.

Our results revealed that in the presence of any RF isotype, patients tend to be younger, and in case of IgA RF, disease duration is significantly shorter as well. The correlation analysis depicts a significant negative correlation with age in SS and SS+pa patients as well. These findings can be at least partially explained by the immune senescence, as highlighted by a recent paper where RF levels were significantly lower in older age groups [20]; however, those patients had RA, while in our SS+RA group, we did not find any significant negative correlation with age. Moreover, when disease activity is higher, the presence of other autoantibodies and inflammatory markers is more frequent in these seropositive cases, which indicate a more severe disease course. Our findings are confirmed by the similar results of a recent study which compared seropositive and seronegative SS patients [21]. These findings emphasize the significance of immunological markers in risk stratification, and they might pave the way towards personalized therapy.

The retrospective nature of the study is one of its limitations. A multi-center design with even more patients could have shown more significant data. Furthermore, repeated measurements of RF isotypes would have allowed us to track longitudinal changes in RF isotypes and further characterize their relation to disease-specific parameters, e.g., the ESSDAI.

## 4. Materials and Methods

We reviewed the data of a total of 164 patients with Sjögren’s syndrome who were regularly followed up at the Division of Clinical Immunology, Faculty of Medicine, University of Debrecen, Hungary. Patients were divided into three groups based on the presence and type of joint involvement. In total, 119 (72.5%) patients were identified to have some kind of inflammatory joint involvement. They were further divided into two groups depending on whether they had polyarthritis as an EGM (SS+pa, *n* = 73; 44.5%) or rheumatoid arthritis associated with Sjögren’s syndrome (SS+RA, *n* = 46; 28%). Patients without any inflammatory joint complaints served as the control group (SS, *n* = 45; 27.4%). Keeping the potentially overlapping joint symptoms of SS+pa and SS+RA in mind, the erosive features of SS+RA joint involvement were verified by imaging techniques. X-rays of the hands, and in some cases, X-rays of the forefeet were performed in all patients with polyarthritis. Moreover, if no erosions were found but clinical suspicion was high, magnetic resonance imaging (MRI) was performed to detect erosions unnoticed by X-ray [3]. The patient groups were compared according to their demographic data, rheumatoid factor isotype levels, further laboratory parameters, disease duration, disease activity, extraglandular manifestations, and associated diseases. To further characterize the connection between immunoglobulin and rheumatoid factor isotypes, patients were re-grouped depending on whether they presented normal or high Ig G, A, and M levels, regardless of the aforementioned subsets.

Laboratory parameters were determined at the Department of Laboratory Medicine. IgM, IgG, and IgA rheumatoid factor levels were determined using enzyme-linked immunosorbent assay (ELISA) reagents from Orgentec Diagnostika GmbH (Mainz, Germany; cat. No.: ORG522G, ORG522M, ORG522A). ELISA kits of Euroimmun AG (Lübeck, Germany) and Svar Life Science AB (Malmö, Sweden) were used for anti-Ro/SS-A (Euroimmun AG, Lübeck, Germany; EA 1595-9601 G), anti-La/SS-B (Euroimmun AG, Lübeck, Germany; EA 1597-9601 G) and anti-CCP (Svar Life Science AB, Malmö, Sweden; ImmunoScan CCPlus, RA-96 PLUS) measurement, respectively. Antinuclear antibodies were detected using indirect immunofluorescence assay on HEp-2 cells (Euroimmun AG, Lübeck, Germany; FC 1520-1010). Routine laboratory tests (turbidimetric rheumatoid factor; total IgG, IgM, and IgA levels; C-reactive protein) were conducted on a Roche Cobas 6000 clinical chemistry analyzer (Roche AG, Basel, Switzerland).

Written informed consent was obtained from each patient. All procedures were conducted in accordance with the Declaration of Helsinki.

### Statistical Analysis

Values are expressed as the mean and standard deviation (SD) or median with interquartile range (IQR) for continuous variables, and frequency is presented as a percentage for categorical variables. Continuous variables were compared with a parametric two-sample *t*-test or nonparametric Mann–Whitney U test for two samples and a Kruskal–Wallis test with an uncorrected Dunn-test for three samples. The effect size was calculated using Cohen’s d (d), r, and η^2^, respectively [22]. Categorical variables were compared with Pearson’s chi-squared test or Fisher’s exact test, and the effect size is reported as Cramér’s V (V). Correlations between variables were calculated using Spearman’s correlation coefficient (r_s_). Sensitivity and specificity were calculated. All statistical tests were two-sided; differences were considered statistically significant at a <0.05 level and were reported using *p*-values and/or 95% confidence intervals (95% CI). Statistical analysis was performed using SPSS Statistics for Windows, version 28.0 (IBM Corp.; Armonk, NY, USA) and GraphPad Prism for Windows, version 10.3.1 (GraphPad Software, Boston, MA, USA).

## 5. Conclusions

Among rheumatoid factor isotypes, IgA and IgM have an additive diagnostic value when the goal is to distinguish between SS+pa and SS+RA; however, they must be combined with total IgG levels. High total IgG levels together with high IgA RF suggest SS, and normal total IgG levels combined with high IgM RF are suggestive of SS+RA. In SS+pa, the most sensitive combination is a high total IgG and normal IgM RF.

Notable, the presence of RF of any isotype predicts a more severe disease course.

Finally, the positive correlation between IgA RF and ESSDAI, the several markers of serological activity of SS, and its negative correlation with age make IgA RF a potential biomarker for early, poor prognosis of SS.

Based on our results, we recommend using IgA RF levels as a complementary marker, especially upon diagnosis or in the development of joint complaints during the disease course of SS patients.

## Figures and Tables

**Table 1:** Demographics and laboratory parameters of the patient groups.

	SS	SS+pa	SS+RA	*p*-Value	Effect Size
Patients (*n* = 164)	45 (27.4%) [20.8–34.9%]	73 (44.5%) [36.8–52.5%]	46 (28%) [21.3–35.6%]		
Women	42 (93.3%)	71 (97.3%)	45 (97.8%)	0.445	V = 0.099
Age (years)	58.9 ± 14.1	60 ± 10.1	63.3 ± 10.8	0.156	η^2^ = 0.023
Disease duration (years)	11 (9–15)	11 (8–19)	13 (10–20)	0.094	η^2^ = 0.017
IgA (g/L)	2.1 (1.6–3)	2.4 (1.7–3.2)	2.2 (1.7–3.2)	0.874	η^2^ = 0.011
IgG (g/L)	13.3 (10.3–19.2) ^3^	11.9 (9.4–17.3) ^3^	10.5 (8.5–11.6) ^1,2^	**0.004**	η^2^ = 0.055
IgM (g/L)	0.9 (0.7–1.6)	1.1 (0.8–1.9)	1 (0.7–1.3)	0.156	η^2^ = 0.011
CRP (mg/L)	2 (0.9–5.3)	2 (1.1–3.9)	2.4 (1.2–5.6)	0.430	η^2^ = 0.002
RF (IU/mL)	26 (14–47)	23 (13–40)	28 (12–89)	0.437	η^2^ = 0.002

Demographics are presented as frequency (%), mean ± SD, or median (IQR). Laboratory parameters are presented as the median (IQR). *p*-Values < 0.05 are highlighted in bold text. ^1^ Significantly different from the SS group. ^2^ Significantly different from the SS+pa group. ^3^ Significantly different from the SS+RA group. Abbreviations: SS: Sjögren’s syndrome patients without joint pain; SS+pa: Sjögren’s syndrome patients with polyarthritis; SS+RA: patients with associated Sjögren’s syndrome and rheumatoid arthritis; IgA: immunoglobulin A antibody; IgG: immunoglobulin G antibody; IgM: immunoglobulin M antibody; CRP: C-reactive protein; and RF: rheumatoid factor.

**Table 2:** Comparison between positive and negative groups of IgA RF, IgG RF, and IgM RF.

	IgA RF				IgG RF				IgM RF			
	Positive (*n* = 89)	Negative (*n* = 75)	*p*	Effect Size	Positive (*n* = 83)	Negative (*n* = 81)	*p*	Effect Size	Positive (*n* = 83)	Negative (*n* = 81)	*p*	Effect Size
***Patient groups***												
SS	29 (32.6)	16 (21.3)	0.108	0.126 ^a^	25 (30.1)	20 (24.7)	0.436	0.061 ^a^	25 (30.1)	20 (24.7)	0.436	0.061 ^a^
SS+pa	35 (39.3)	38 (50.7)	0.145	0.114 ^a^	33 (39.8)	40 (49.4)	0.215	0.097 ^a^	33 (39.8)	40 (49.4)	0.215	0.097 ^a^
SS+RA	25 (28.1)	21 (28)	0.990	0.001 ^a^	25 (30.1)	21 (25.9)	0.550	0.047 ^a^	25 (30.1)	21 (25.9)	0.550	0.047 ^a^
***Demographics***												
Age (years)	56.8 ± 11.9	65.2 ± 9.3	**<0.001**	0.775 ^b^	57.4 ± 12.7	63.9 ± 9.3	**<0.001**	0.580 ^b^	58 ± 12.4	63.2 ± 10.1	**0.004**	0.457 ^b^
Disease duration (years)	11 (8–15)	13 (10–21)	**0.013**	0.193 ^c^	13 (9–20)	13 (9–20)	0.217	0.096 ^c^	11 (8–18)	12 (9–19)	0.349	0.073 ^c^
***Disease-specific data***												
Time to joint symptoms (years)	0 (−1–2)	0 (0–2)	0.465	0.067 ^c^	0 (−0.5–2)	0 (−0.5–2)	0.738	0.031 ^c^	0 (−1–2)	0 (−1–2)	0.952	0.006 ^c^
DAS28 (score)	3 (2–3)	2 (2–3)	0.051	0.288 ^c^	2 (2–3)	3 (2–3)	0.843	0.029 ^c^	2 (2–3)	3 (2–3)	0.869	0.024 ^c^
ESSDAI (score)	2 (1–4)	1 (0–2)	**<0.001**	0.272 ^c^	2 (1–4)	1 (0–2)	**0.005**	0.222 ^c^	2 (1–4)	1 (0–2)	**0.028**	0.171 ^c^
***Laboratory parameters***												
Initial anti-CCP (+)	23 (26.1)	8 (10.7)	**0.012**	0.196 ^a^	21 (25.6)	10 (12.3)	**0.031**	0.169 ^a^	22 (26.8)	9 (11.1)	**0.011**	0.200 ^a^
Last anti-CCP (+)	21 (23.6)	11 (14.7)	0.151	0.112 ^a^	22 (26.5)	10 (12.3)	**0.022**	0.179 ^a^	22 (26.5)	10 (12.3)	**0.022**	0.179 ^a^
RF (+)	82 (92.1)	38 (50.7)	**<0.001**	0.466 ^a^	75 (90.4)	45 (55.6)	**<0.001**	0.393 ^a^	78 (94)	42 (51.9)	**<0.001**	0.475 ^a^
anti-Ro/SS-A (+)	70 (78.7)	22 (29.3)	**<0.001**	0.495 ^a^	54 (65.1)	38 (46.9)	**0.019**	0.183 ^a^	55 (66.3)	37 (45.7)	**0.008**	0.207 ^a^
anti-La/SS-B (+)	55 (61.8)	11 (14.7)	**<0.001**	0.479 ^a^	45 (54.2)	21 (25.9)	**<0.001**	0.288 ^a^	49 (59)	17 (21)	**<0.001**	0.388 ^a^
ANA (+)	80 (89.9)	37 (49.3)	**<0.001**	0.447 ^a^	66 (79.5)	51 (63)	**0.019**	0.183 ^a^	69 (83.1)	48 (59.3)	**0.001**	0.264 ^a^
CRP (high)	26 (29.2)	16 (21.3)	0.249	0.090 ^a^	23 (27.7)	19 (23.5)	0.533	0.049 ^a^	23 (27.7)	19 (23.5)	0.533	0.049 ^a^
ESR (high)	44 (49.4)	20 (26.7)	**0.003**	0.233 ^a^	45 (54.2)	19 (23.5)	**<0.001**	0.315 ^a^	44 (53)	20 (24.7)	**<0.001**	0.290 ^a^
WBC (normal)	54 (60.7)	54 (72)	0.128	0.119 ^a^	50 (60.2)	58 (71.6)	0.125	0.120 ^a^	51 (61.4)	57 (70.4)	0.228	0.094 ^a^
IgA (g/L)	2.8 (2.0–3.6)	1.9 (1.4–2.5)	**<0.001**	0.345 ^c^	2.8 (2.0–3.5)	1.9 (1.4–2.5)	**<0.001**	0.316 ^c^	2.7 (1.9–3.5)	1.9 (1.4–2.6)	**<0.001**	0.276 ^c^
IgG (g/L)	14.7 (11.1–19.5)	10.3 (8.7–11.8)	**<0.001**	0.426 ^c^	16.2 (10.9–19.7)	10.3 (8.5–11.8)	**<0.001**	0.454 ^c^	14.4 (10.7–19.7)	10.4 (8.5–12)	**<0.001**	0.394 ^c^
IgM (g/L)	1.09 (0.7–1.54)	0.97 (0.65–1.82)	0.784	0.021 ^c^	1.14 (0.9–1.63)	0.87 (0.63–1.54)	**0.008**	0.207 ^c^	1.14 (0.88–1.84)	0.87 (0.58–1.47)	**0.003**	0.232 ^c^

Values are presented as frequency (%), mean ± SD, or median (IQR). *p*-values < 0.05 are highlighted in bold. ^a^ Effect size reported as V. ^b^ Effect size reported as d. ^c^ Effect size reported as r. Reference range of laboratory parameters: IgA RF < 20 U/mL, IgG RF < 20 U/mL, IgM RF < 20 U/mL, anti-CCP < 25 U/mL, RF < 14 IU/mL, anti-Ro/SS-A < 10 U/mL, anti-La/SS-B < 10 U/mL, CRP < 5.2 mg/L for male and <4.6 mg/L for female, ESR < 20 mm/h, WBC: 4.8–10.8 G/L. Abbreviations: *p*: *p*-value; IgA RF: immunoglobulin A isotype of rheumatoid factor; IgG RF: immunoglobulin G isotype of rheumatoid factor; IgM RF: immunoglobulin M isotype of rheumatoid factor; SS: Sjögren’s syndrome patients without joint pain; SS+pa: Sjögren’s syndrome patients with polyarthritis; SS+RA: patients with associaton of Sjögren’s syndrome and rheumatoid arthritis; DAS28: Disease Activity Score-28 for Rheumatoid Arthritis; ESSDAI: EULAR Sjögren’s syndrome disease activity index; anti-CCP: anti-cyclic citrullinated peptide antibody; RF: rheumatoid factor; anti-Ro/SS-A: anti–Sjögren’s-syndrome-related antigen A antibody; anti-La/SS-B: anti–Sjögren’s-syndrome-related antigen B antibody; ANA: antinuclear antibody; CRP: C-reactive protein; ESR: erythrocyte sedimentation rate; and WBC: white blood cell.

**Table 3:** Drugs used for the disease management in positive and negative groups of IgA RF, IgG RF, and IgM RF.

	IgA RF				IgG RF				IgM RF			
	Positive (*n* = 89)	Negative (*n* = 75)	*p*	Effect Size (V)	Positive (*n* = 83)	Negative (*n* = 81)	*p*	Effect Size (V)	Positive (*n* = 83)	Negative (*n* = 81)	*p*	Effect Size (V)
Methylprednisolone	48 (53.9)	31 (41.3)	0.108	0.126	42 (50.6)	37 (45.7)	0.528	0.049	42 (50.6)	37 (45.7)	0.528	0.049
Methotrexate	24 (27)	15 (20)	0.297	0.082	21 (25.3)	18 (22.2)	0.643	0.036	20 (24.1)	19 (23.5)	0.923	0.008
Leflunomide	11 (12.4)	7 (9.3)	0.537	0.048	11 (13.3)	7 (8.6)	0.345	0.074	10 (12)	8 (9.9)	0.656	0.035
Sulfasalazine	2 (2.2)	7 (9.3)	0.081	0.155	1 (1.2)	8 (9.9)	**0.017**	0.190	2 (2.4)	7 (8.6)	0.097	0.137
Azathioprine	4 (4.5)	4 (5.3)	1.000	0.019	5 (6)	3 (3.7)	0.720	0.054	5 (6)	3 (3.7)	0.720	0.054
Chloroquine	30 (33.7)	22 (29.3)	0.549	0.047	25 (30.1)	27 (33.3)	0.658	0.034	27 (32.5)	25 (30.9)	0.819	0.018
Certolizumab	3 (3.4)	3 (4)	1.000	0.017	5 (6)	1 (1.2)	0.210	0.128	5 (6)	1 (1.2)	0.210	0.128
Tocilizumab	2 (2.2)	1 (1.3)	1.000	0.034	2 (2.4)	1 (1.2)	1.000	0.044	3 (3.6)	0 (0)	0.246	0.135

Values are presented as frequency (%). *p*-values < 0.05 are highlighted in bold. Reference range of laboratory parameters: IgA RF < 20 U/mL, IgG RF < 20 U/mL, IgM RF < 20 U/mL. Abbreviations: *p*: *p*-value; IgA RF: immunoglobulin A isotype of rheumatoid factor; IgG RF: immunoglobulin G isotype of rheumatoid factor; and IgM RF: immunoglobulin M isotype of rheumatoid factor.

**Table 4:** Co-occurrence of certain IgG and IgARF/IgMRF characteristics in the patient groups.

	SS (*n* = 45)	SS+pa (*n* = 73)	SS+RA (*n* = 46)	*p*-Value	Effect Size (V)
High total IgG + high IgA RF	18 (40%) ^2,3^	19 (26%) ^1^	8 (17.4%) ^1^	**0.050**	0.191
Specificity (95% CI)	77.3 (69–83.9)	71.4 (61.4–79.7)	68.6 (59.8–76.3)		
Sensitivity (95% CI)	40 (27–54.6)	26 (17.3–37.1)	17.4 (9.1–30.7)		
Normal total IgG + high IgM RF	7 (15.6%) ^3^	15 (20.5%) ^3^	17 (37%) ^1,2^	**0.039**	0.199
Specificity (95% CI)	73.1 (64.5–80.3)	73.6 (63.8–81.6)	81.4 (73.4–87.4)		
Sensitivity (95% CI)	15.6 (7.7–28.8)	20.6 (12.9–31.2)	37 (24.5–51.4)		
High total IgG + normal IgM RF	4 (8.9%)	9 (12.3%)	1 (2.2%)	0.128	0.162
Specificity (95% CI)	91.6 (85.2–95.4)	94.5 (87.8–97.6)	89 (82.1–93.5)		
Sensitivity (95% CI)	8.9 (3.5–20.7)	12.3 (6.6–21.8)	2.2 (0.1–11.3)		

Abbreviations: SS: Sjögren’s syndrome patients without joint pain; SS+pa: Sjögren’s syndrome patients with polyarthritis; SS+RA: patients with Sjögren’s syndrome associated with rheumatoid arthritis; IgA: immunoglobulin A; IgG: immunoglobulin G; IgM: immunoglobulin M; RF: rheumatoid factor; and CI: confidence interval. ^1^ Significantly different from the SS group ^2^ Significantly different from the SS+pa group. ^3^ Significantly different from the SS+RA group. *p*-values < 0.05 are highlighted in bold.

## Data Availability

Dataset available upon request from the authors.

## Abbreviations

The following abbreviations are used in this manuscript:

AID	Autoimmune disease
ANA	Antinuclear antibody
CCP	Cyclic citrullinated peptide
DAS28	Disease activity score
EGM	Extraglandular manifestation
ELISA	Enzyme-linked immunosorbent assay
ESR	Erythrocyte sedimentation rate
ESSDAI	EULAR Sjögren’s syndrome disease activity index
EULAR	European Alliance of Associations for Rheumatology
HLA	Human leukocyte antigen
Ig	Immunoglobulin
IQR	Interquartile range
MRI	Magnetic resonance imaging
pa	Polyarthritis
RA	Rheumatoid arthritis
RF	Rheumatoid factor
SD	Standard deviation
SS	Sjögren’s syndrome
